# Association mapping in multiple yam species (*Dioscorea* spp.) of quantitative trait loci for yield-related traits

**DOI:** 10.1186/s12870-023-04350-4

**Published:** 2023-07-11

**Authors:** I.I. Adejumobi, Paterne A. Agre, A.S. Adewumi, T.E. Shonde, I.M. Cipriano, J.L. Komoy, J.G. Adheka, D.O. Onautshu

**Affiliations:** 1 Department of Biotechnology, Faculty of Science, University of Kisangani, Kisangani, DR Congo; 2 International Institute of Tropical Agriculture, Lagos, Nigeria

**Keywords:** DRC, Genome-wide association study, Mixed linear model, Population structure, Putative genes

## Abstract

**Background:**

Yam (*Dioscorea* spp.) is multiple species with various ploidy level and considered as cash crop in many producing areas. Selection based phenotyping for yield and its related traits such as mosaic virus and anthracnose diseases resistance and plant vigor in multiple species of yam is lengthy however, marker information has proven to enhance selection efficiency.

**Methodology:**

In this study, a panel of 182 yam accessions distributed across six yam species were assessed for diversity and marker-traits association study using SNP markers generated from Diversity Array Technology platform. For the traits association analysis, the relation matrix alongside the population structure were used as co-factor to avoid false discovery using Multiple random Mixed Linear Model (MrMLM) followed by gene annotation.

**Results:**

Accessions performance were significantly different (*p* < 0.001) across all the traits with high broad-sense heritability (H^2^). Phenotypic and genotypic correlations showed positive relationships between yield and vigor but negative for yield and yam mosaic disease severity. Population structure revealed k = 6 as optimal clusters-based species. A total of 22 SNP markers were identified to be associated with yield, vigor, mosaic and anthracnose diseases resistance. Gene annotation for the significant SNP loci identified some putative genes associated with primary metabolism, pest and resistance to anthracnose disease, maintenance of NADPH in biosynthetic reaction especially those involving nitro-oxidative stress for resistance to mosaic virus, and seed development, photosynthesis, nutrition use efficiency, stress tolerance, vegetative and reproductive development for tuber yield.

**Conclusion:**

This study provides valuable insights into the genetic control of plant vigor, anthracnose, mosaic virus resistance, and tuber yield in yam and thus, opens an avenue for developing additional genomic resources for markers-assisted selection focusing on multiple yam species.

**Supplementary Information:**

The online version contains supplementary material available at 10.1186/s12870-023-04350-4.

## Introduction

Yam is among the principal root and tuber crops, including cassava and potato, that are widely grown and consumed as subsistence staples in sub-Saharan Africa where over 90% of the global production resides [1–3]. It is a group of multi-species monocot with X = 20 as the basic chromosome number and cultivated for the starchy underground tubers and aerial bulbils in the yam belts of west and central Africa [2]. In the Democratic Republic of Congo (DRC), yams play an important role in ensuring the sustenance of food security, primarily for the rural populace [4]. DRC is home to a few species of yam which includes *D. alata* (water/greater yam)*, D. cayenensis* (yellow Guinea yam)*, D. dumetorum* (bitter yam), and *D. rotundata* (white Guinea yam), *D. bulbifera* (aerial yam), *D. burkilliana* (wild yam), and *D. praehensilis* (bush yam) [4–6]. Though, *D. alata, D. cayenensis* and *D. rotundata* are widely cultivated among the farmers compared to other species [4, 7].

Despite the contribution of yam to the rural sustenance in DRC, production is seasonally met with several constraints including but not limited to poor agronomic performance (yield and related traits) and major pathological issues namely yam mosaic disease (YMD) and yam anthracnose disease (YAD). These constraints have consistently affected the performance of many cultivated landraces and thus, aggravating the loss of interest in yam production in many producing areas [4]. YMD is caused by yam mosaic viruses (YMV) while YAD is caused by *Colletotrichum glo- eosporioides* (Penz.). Percentage yield loss attributable to the synergy effect of these diseases have been reported to be above 50% [8, 9]. Developing and delivery of new and improved varieties for improved vigor and yield potential as well as better tolerance to YMD and YAD could increase the productivity of resource-poor farmers characterized by low use of external farm inputs.

The art of breeding for improved varieties demands a thorough understanding of the genetic basis of the traits. However, the lack of information on the genetic diversity as well as the genetic architecture of key and economic traits have been a major hindrance to the success of improved cultivar development in DRC. Findings from other yam producing regions have reported the influence of quantitative inheritance for key and economic traits in yam [10, 11]. Genome-Wide Association Studies (GWAS) is an ideal method for dissecting the genetic control of complex traits as it uses historic recombination events accumulated over many generations. GWAS has been successfully used to genetically dissect yam traits of economic importance such as tuber yield and mosaic virus resistance in *D. rotundata* [10], sex determination and cross compatibility in *D. alata* [11], and tuber dry matter content and oxidative browning in *D. alata* [12]. These studies have shown the importance of GWAS in identifying genomic regions and candidate genes associated with key and economic traits in yam, however they have been species-specific and thus, very likely that the impact of the finding from one species may not be perfectly transferrable to another species. This is due to pre and post-zygote challenges previously reported from crosses originating from different yam species [11]. Thus, identifying genomic regions associated with economic traits of importance in multiple yam species could offer a better breeding impact. This remained an area that has not been considered and exploited as it has been done for crops with multiple species. Realizing this would facilitate the development of molecular markers that can be relied upon for early generation traits selection in multiple species of *Dioscorea*.

As a contribution to further improve upon this method of breeding, identifying genomics regions associated with yield and related traits for possible markers development for selection in multiple yam species will offer an advantage over the current system of species-specific markers. The objective of this study was to dissect the genetic control of tuber yield and related traits (vigor, YMD, and YAD) in a panel of yam consisting of six species.

## Results

### Phenotypic variation, correlation, and heritability among the 182 yam accessions

**Fig. 1:**
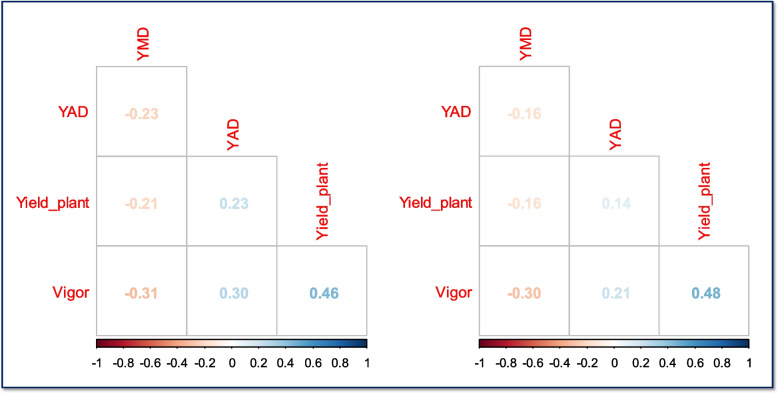
Genotypic (left) and phenotypic (right) and relationships among traits. *Vigor* Plant vigor, *YAD* Yam anthracnose severity, *YMD* Yam mosaic severity, Yield_plant Tuber yield per plant

Significant interaction effect of year by accession was observed at *p* < 0.05 for yield and *p* < 0.001 for other parameters. Accession effect was significant at *p* < 0.001 for all the traits while year effect was significant for YAD at *p* < 0.01 and YMD severity at *p* < 0.001 (Table 1). GCV estimate ranged from moderate classification for YMV, YAD, and vigor (between 10 and 20%) to high classification in yield (43.71%). PCV estimate ranged from moderate classification for YMD and YAD (16.22 and 17.03, respectively) to high classification for yield and vigor (52.32 and 20.75, respectively). H^2^ estimate was high for all the characters (Table 1). Both phenotypic and genotypic correlations revealed positive relationship between yield and plant vigor. YMD had negative relationship with plant vigor however, YAD had positive correlation with plant vigor (Fig. 1).

**Table 1:** Estimates of variance, coefficients of variation and heritability in a panel of 182 yam accessions

Source	DF	Vigor	YAD	YMD	Yield
Species	5	0.52**	25.37***	1.64***	6.44***
Year	1	0.19	13.16**	11.97***	0.28
Accession	177	0.75***	2.79***	2.15***	3.91***
Species * Year	5	0.19	0.46	0.07	0.08
Year * Accession	177	0.27***	1.13***	0.47***	1.23*
Residual	335	0.13	0.53	0.31	0.97
CV%		15.95	11.91	10.43	48.6
Mean		2.31	6.3	5.23	2.02
δ^2^g		0.16	0.86	0.6	0.89
δ^2^p		0.23	1.15	0.72	1.20
GCV (%)		17.31	14.73	14.8	46.70
PCV (%)		20.75	17.03	16.22	54.23
H^2^ (%)		69.00	74.34	83.93	74.10

*DF* Degree of freedom, *Vigor* Plant vigor, *YAD* Yam anthracnose disease, *YMD* Yam mosaic disease, *Yield* Tuber yield per plant, *CV* Coefficient of variation, δ^2^g Genotypic variance, δ^2^p Phenotypic variance, *GCV* Genotypic coefficient of variability, *PCV* Phenotypic coefficient of variability, H^2^ Heritability

### Summary statistics and genetic diversity assessment

A total number of 20,275 SNPs was generated by the DArTseq protocol from which 11,722 were retained after filtering for MAF, maximum missing, genotype quality, and read depth. MAF varied from 0.052 to 0.50 with an average of 0.231, gene diversity varied from 0.09 to 0.50 with an average of 0.324, and the observed heterozygosity varied from 0 to 0.576 with an average of 0.254. The polymorphic information content varied from 0.09 to 0.375 with an average of 0.264.

**Fig. 2:**
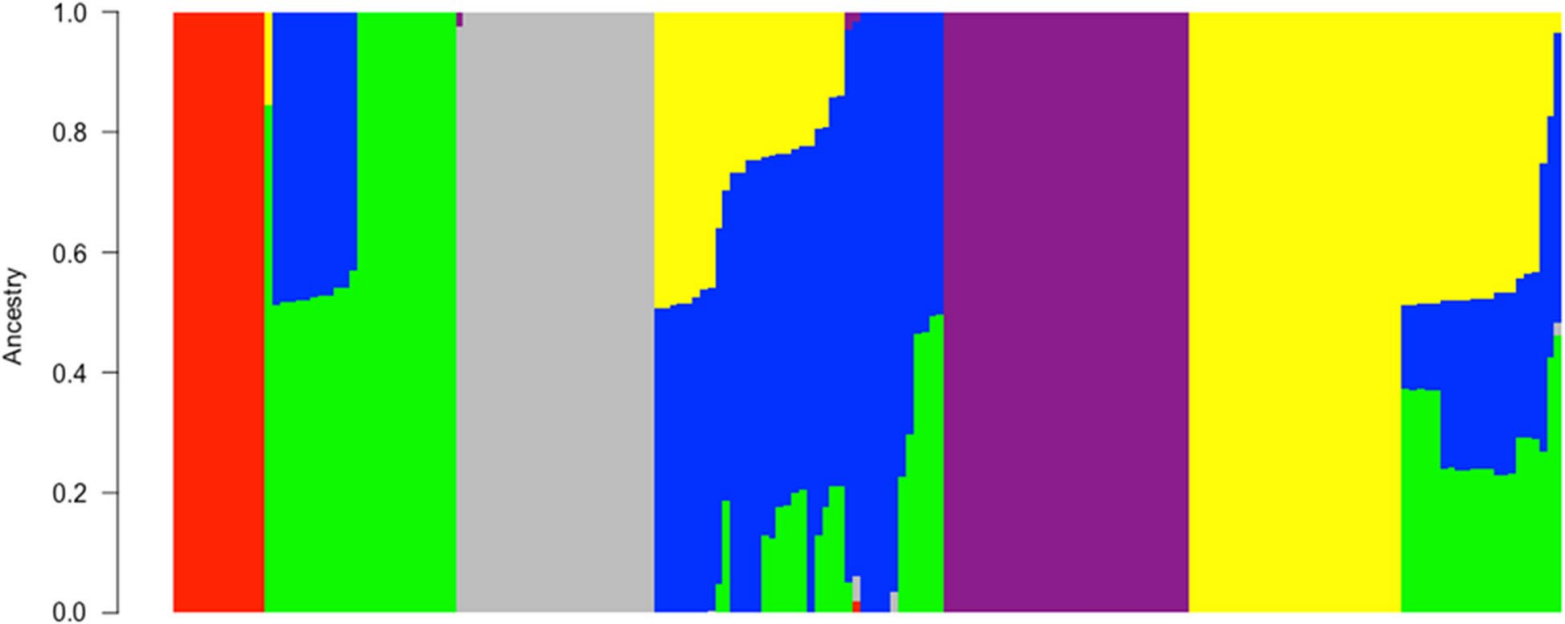
Graphical representation of yam accessions population structure based on admixture analysis. Populations were set at k = 6. The colors represent the six groups: group 1 (red), group 2 (green), group 3 (gray), group 4 (blue), group 5 (purple), and group 6 (yellow) based on a membership coefficient of ≥ 50%

The population structure analysis revealed cluster K = 6 (Fig. 2; Sup. Figure 1) as optimal cluster number. Approximately 89% of the yam accessions were successfully assigned to at least one of the clusters while 11% distributed across four species (Sup. Table 1) were considered as admixt with assigned probability less than 0.5.

**Fig. 3:**
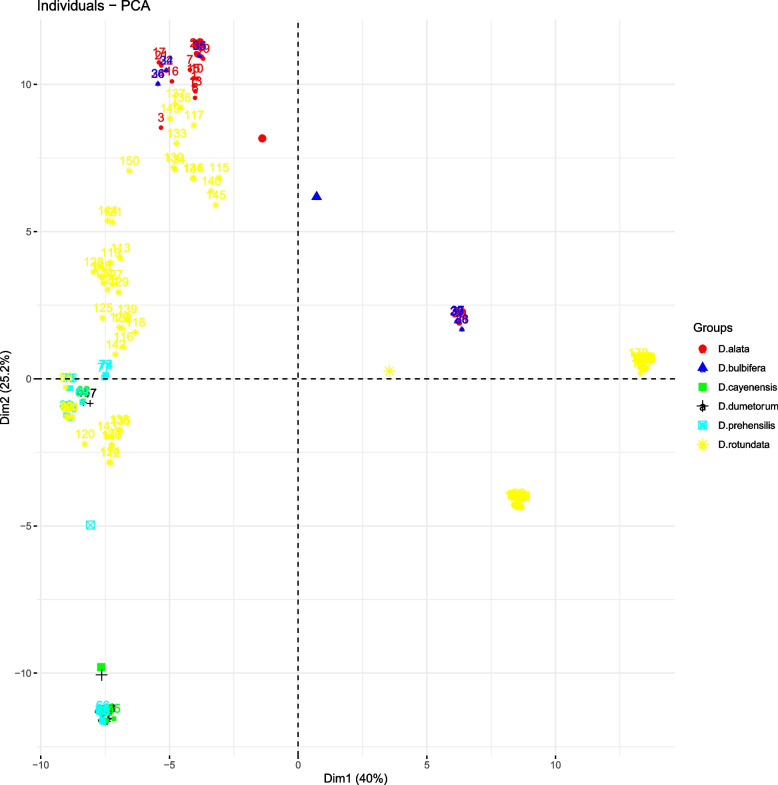
Species-based scatter plot of yam accessions using 11,722 SNP markers. Each color represents the species, and each dot represents the individual within the species

**Fig. 4:**
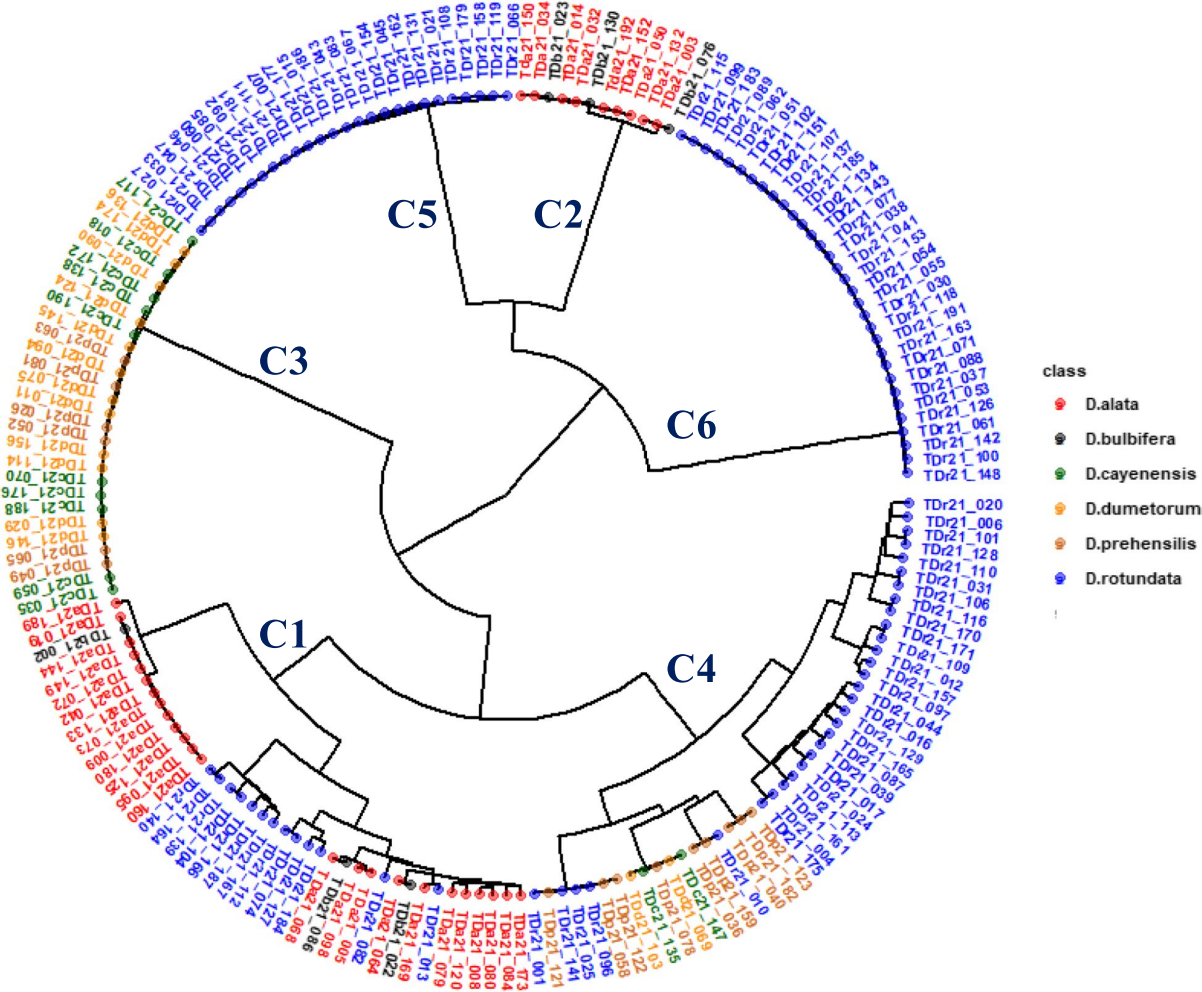
Phylogeny diagram of the panel of 182 yam accessions based on yam species as a co-factor

Exploring the genetic relationship through principal component analysis showed that the first two PCs account for 65.2% of the total variation. The species-based PCA plot revealed a segregation plot of the six yam species except for some few cases where we observed some possible mixture in *D. alata* and *D. bulbifera* as well as in *D. praehensilis* and *D. rotundata* (Fig. 3). The species pairwise differentiation showed that the genomes of *D. cayenensis* and *D. bulbifera* are the most distantly related (0.768, *p* < 0.001) while the genomes of *D. alata* and *D. bulbifera* were the most related (0.016, *p* < 0.038) (Table 2). The genome relatedness of *D. alata* and *D. bulbifera* was further confirmed by the phylogeny analysis that grouped both species into the same clusters (Fig. 4).

**Table 2:** Pairwise species differentiation among 182 accessions of yam landraces

Species	*D. ala*	*D. bulb*	*D. cay*	*D. dum*	*D. prae*	*D. rot*
*D. alata*	-	0.038	0.001	0.001	0.001	0.001
*D. bulbifera*	0.016	-	0.001	0.001	0.001	0.003
*D. cayenensis*	0.710	0.768	-	0.308	0.009	0.001
*D. dumetorum*	0.694	0.744	0.074	-	0.021	0.001
*D. praehensilis*	0.528	0.530	0.215	0.182	-	0.001
*D. rotundata*	0.374	0.337	0.539	0.524	0.373	-

D. ala. D. alata, D. bulb. D. bulbifera, D. cay. D. cayenensis, D. dum. D. dumetorum, D. prae. D. praehensilis, D. rot. D. rotundata

The phylogeny diagram revealed six genetic groups or clusters (Fig. 4). The first genetic group has the 40 members distributed across species of *D. alata* (60%), *D. bulbifera* (7.5%), and *D. rotundata* (32.5%). This group has genetic distance ranging from 0.006 to 0.345 with an average of 0.269. The second cluster has 12 members with genetic distance ranging from 0.008 to 0.339 with an average of 0.302. This cluster has the least number of membership and distributed across species of *D. alata* (75%) and *D. bulbifera* (25%). The third cluster has 28 members with genetic distance ranging from 0.005 to 0.345 with an average of 0.251. *D. cayenensis* (35.7%), *D. dumetorum* (42.9%), and *D. praehensilis* (21.4%) were identified in this cluster. The fourth cluster has 44 members with genetic distance ranging from 0.008 to 0.344 with an average of 0.261. The cluster has the largest cluster members and distributed across species of *D. cayenensis* (4.5%), *D. dumetorum* (4.5%), *D. praehensilis* (20.5%), and *D. rotundata* (70.45%). The fifth cluster has 26 members with genetic distance ranging from 0.006 to 0.332 with an average of 0.264. The members of this cluster belongs to *D. rotundata*. The sixth cluster has 32 members with genetic distance ranging from 0.005 to 0.343 with an average of 0.263. The members of this cluster also belong to *D. rotundata* (Fig. 4)*.*

### Genome-wide scan for traits

#### Plant vigor, yam anthracnose severity, yam mosaic severity and tuber yield

**Fig. 5:**
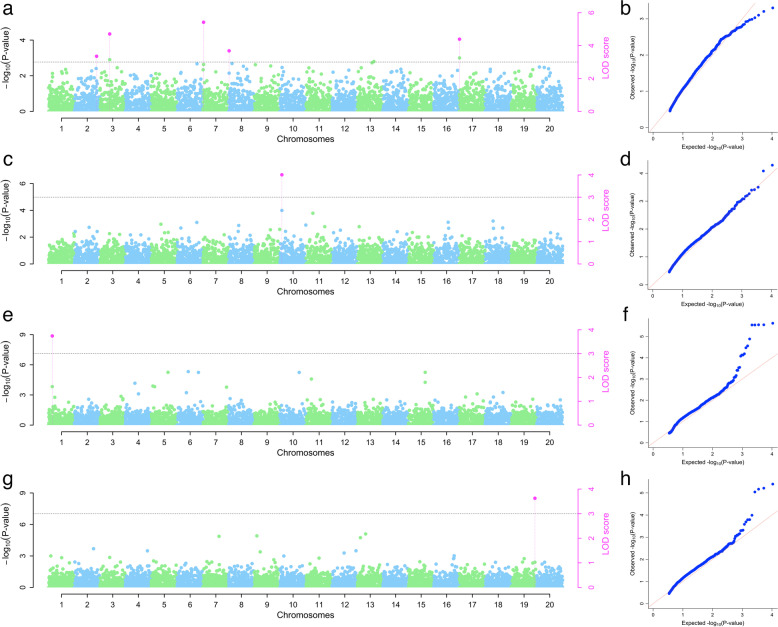
Genome-wide association analysis of plant vigor (vigor), yam anthracnose severity (YAD), yam mosaic severity (YMD), and tuber yield per plant (yield) for the evaluation year 2021. Manhattan and QQ plots indicating SNPs associated with the vigor (3a and b), YAD (3c and d), YMD (3e and f), and yield (3 g and h). The y-axis represents the p-value of the marker-trait association on a –log10 scale

In 2021, the GWAS results revealed five significant SNP markers on chromosomes 2, 3, 7, 8, and 17 associating with plant vigor with LOD values ranging from 3.35 to 5.42, MAF ranging from 0.07 to 0.45, and the marker chr_7356 explained the highest phenotypic variance (63%). For anthracnose severity, one SNP marker was found on chromosome 10 with LOD value of 4.01, MAF of 0.17 and explained 19% of the phenotypic variance. For mosaic severity, one SNP marker was found on chromosome 1 with LOD value of 3.74, MAF of 0.16 and explained 18% of the phenotypic variance. For tuber yield, one SNP marker was found on chromosome 19 with LOD value of 3.63, MAF of 0.29 and explained less than 1% of the phenotypic variance (Table 3 and Fig. 5).

**Table 3:** SNP markers associated with plant vigor, yam anthracnose severity, yam mosaic severity, and tuber yield

Year	Trait name	Method	SNP marker	Chr	Marker position (bp)	QTN effect	LOD score	R2 (%)	MAF	Favorable alleles
2021	Vigor	mrMLM	chr_3_7386	3	7386	-0.46	4.71	3.34	0.21	T
	Vigor	mrMLM	chr_7_356	7	356	-0.62	5.42	62.98	0.45	A
	Vigor	FASTmrEMMA	chr_17_171	17	171	0.94	4.39	15.82	0.13	A
	Vigor	pKWmEB	chr_2_41285	2	41,285	2.3E-05	3.35	2.97	0.15	G
	Vigor	pKWmEB	chr_8_297	8	297	1.9E-05	3.68	0.00	0.07	A
	YAD	FASTmrEMMA	chr_10_1122	10	1122	49.34	4.01	18.66	0.17	C
	YMD	FASTmrEMMA	chr_1_1994	1	1994	43.47	3.74	18.01	0.16	A
	Yield	pLARmEB	chr_19_56351	19	56,351	3.0E-05	3.63	0.00	0.29	A
2022	Vigor	mrMLM	chr_17_10919	17	10,919	0.24	4.79	13.26	0.44	C
	Vigor	FASTmrMLM	chr_20_15436	20	15,436	-0.23	5.51	8.36	0.30	T
	Vigor	pLARmEB	chr_7_107712	7	107,712	0.00	3.09	0.00	0.48	A
	Vigor	pLARmEB	chr_8_1451	8	1451	-0.13	4.21	1.96	0.32	T
	Vigor	pKWmEB	chr_8_1451	8	1451	-0.13	3.22	2.74	0.32	T
	YAD	mrMLM	chr_1_732	1	732	14.93	3.64	7.51	0.37	G
	YAD	mrMLM	chr_4_3941	4	3941	13.15	4.66	10.74	0.35	G
	YAD	mrMLM	chr_14_676	14	676	41.08	7.56	3.05	0.05	A
	YAD	FASTmrMLM	chr_13_4907	13	4907	-14.72	3.87	1.16	0.27	C
	YAD	FASTmrMLM	chr_15_72867	15	72,867	31.59	8.57	5.35	0.10	C
	YAD	FASTmrEMMA	chr_14_676	14	676	65.04	4.74	9.51	0.06	A
	YAD	FASTmrEMMA	chr_20_12730	20	12,730	45.21	4.45	8.64	0.12	T
	YAD	pLARmEB	chr_8_49184	8	49,184	14.49	4.15	3.49	0.31	C
	YAD	pLARmEB	chr_15_72867	15	72,867	31.13	4.69	3.26	0.10	C
	YAD	pKWmEB	chr_8_49184	8	49,184	14.49	4.48	7.61	0.31	C
	YAD	pKWmEB	chr_15_72867	15	72,867	31.13	7.46	6.09	0.10	C
	YMD	mrMLM	chr_2_82593	2	82,593	17.01	4.63	5.48	0.14	T
	YMD	mrMLM	chr_15_19801	15	19,801	22.87	9.69	13.04	0.21	T
	YMD	FASTmrMLM	chr_2_11441	2	11,441	-12.94	6.38	15.38	0.36	A
	YMD	FASTmrEMMA	chr_2_11441	2	11,441	-23.68	6.16	12.08	0.36	A
	YMD	pLARmEB	chr_15_19801	15	19,801	14.91	6.12	5.39	0.21	T
	YMD	pKWmEB	chr_2_11441	2	11,441	-12.94	6.20	18.24	0.36	A
	Yield	FASTmrEMMA	chr_8_39074	8	39,074	18.49	3.48	7.73	0.14	T
	Yield	FASTmrEMMA	chr_16_47690	16	47,690	-53.80	5.15	42.96	0.08	C
	Yield	pLARmEB	chr_20_13840	20	13,840	4.34	4.08	6.10	0.34	T
Combined	Vigor	FASTmrMLM	chr_1_9881	1	9881	-1.8E-05	3.60	1.0E-07	0.43	G
	Vigor	pKWmEB	chr_2_41285	2	41,285	-1.0E-04	11.18	2.41	0.15	G
	Vigor	pKWmEB	chr_5_42	5	42	2.7E-05	5.26	0.00	0.11	C
	Vigor	pKWmEB	chr_6_17975	6	17,975	1.4E-05	3.41	0.00	0.05	G
	Vigor	pKWmEB	chr_8_1451	8	1451	0.00	3.70	2.13	0.32	T
	Vigor	pKWmEB	chr_8_297	8	297	2.7E-05	10.57	0.00	0.07	A
	Vigor	pKWmEB	chr_12_31668	12	31,668	0.00	3.73	23.07	0.48	C
	Vigor	pKWmEB	chr_12_19414	12	19,414	0.00	5.09	0.28	0.19	A
	Vigor	pKWmEB	chr_14_13604	14	13,604	2.0E-05	3.48	1.69	0.22	A
	Vigor	pKWmEB	chr_15_2528	15	2528	-1.74E-05	4.49	0.16	0.11	G
	Vigor	pKWmEB	chr_17_868	17	868	-1.84E-05	3.13	0.05	0.31	A
	Vigor	pKWmEB	chr_18_14253	18	14,253	-2.22E-05	5.69	1.93	0.43	A
	Vigor	pKWmEB	chr_20_8092	20	8092	-5.96E-05	5.17	0.50	0.20	C
	YAD	FASTmrMLM	chr_10_130470	10	130,470	15.28	3.75	0.00	0.14	C
	YAD	FASTmrEMMA	chr_16_14899	16	14,899	-49.27	3.53	10.66	0.11	A
	YAD	pLARmEB	chr_6_2060	6	2060	-8.55E-05	3.14	1.3E-10	0.28	A
	YAD	pLARmEB	chr_10_130470	10	130,470	15.28	3.36	0.00	0.14	C
	YAD	pKWmEB	chr_10_130470	10	130,470	13.04	3.34	0.00	0.14	C
	YMD	mrMLM	chr_1_1994	1	1994	32.25	5.58	2.65	0.16	A
	YMD	mrMLM	chr_20_10315	20	10,315	29.43	4.35	55.44	0.19	A
	YMD	FASTmrMLM	chr_15_19801	15	19,801	38.58	6.73	39.65	0.21	T
	YMD	FASTmrEMMA	chr_15_19801	15	19,801	76.25	6.24	55.80	0.21	T
	YMD	pLARmEB	chr_15_19801	15	19,801	38.58	6.73	18.40	0.21	T
	Yield	FASTmrEMMA	chr_8_39074	8	39,074	15.58	3.31	6.94	0.14	T
	Yield	FASTmrEMMA	chr_9_3704	9	3704	-46.98	5.28	41.46	0.08	A
	Yield	FASTmrEMMA	chr_13_43659	13	43,659	9.51	3.40	3.79	0.13	A

*Chr* Chromosome, r^2^ Total phenotypic variance explained, *LOD* Logarithm of Score *MAF* Minor allele frequency, Vigor Plant vigor, *YAD* Yam anthracnose severity, *YMD* Yam mosaic severity, Yield Tuber yield per plant

**Fig. 6:**
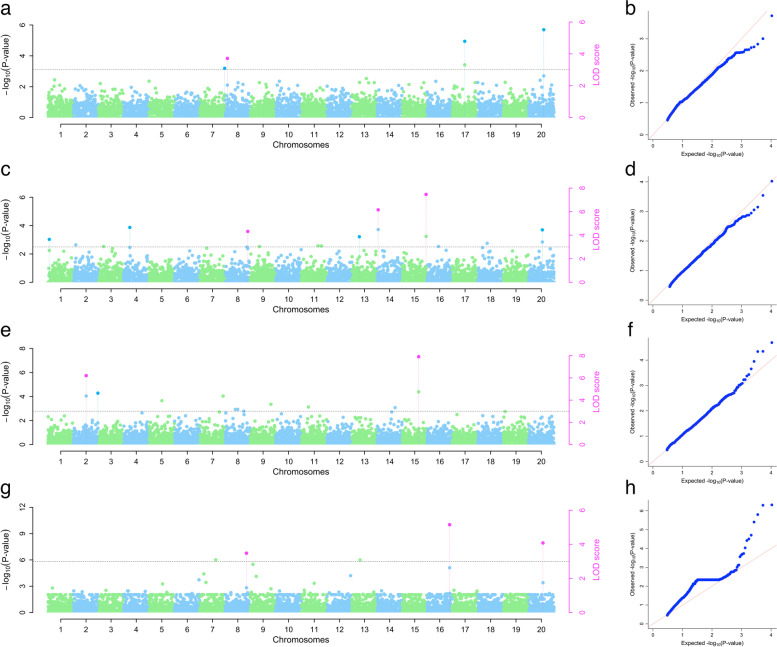
Genome-wide association analysis of plant vigor (vigor), yam anthracnose severity (YAD), yam mosaic severity (YMD), and tuber yield per plant (yield) for the evaluation year 2022. Manhattan and QQ plots indicating SNPs associated with the vigor (4a and b), YAD (4c and d), YMD (4e and f), and yield (4 g and h). The y-axis represents the p-value of the marker-trait association on a –log10 scale

In 2022, five significant SNP markers on chromosomes 7, 8, 17, and 20 were associated with plant vigor with LOD values ranging from 3.01 to 5.51, MAF ranging from 0.32 to 0.48, and the marker chr_17_10919 explained the highest phenotypic variance (13%). For anthracnose severity, eleven significant SNP markers were found on chromosomes 1, 4, 8, 13, 14, 15, and 20 with LOD values ranging from 3.64 to 8.57, MAF ranging from 0.05 to 0.37, and the marker chr_4_3941 explained the highest phenotypic variance (11%). For mosaic severity, six significant SNP markers were found on chromosomes 2 and 15 with LOD values ranging from 4.63 to 9.69, MAF ranging from 0.14 to 0.36, and the marker chr_2_11441 explained the highest phenotypic variance (18%). For tuber yield, three significant SNP markers were found on chromosomes 6, 8, and 20 with LOD values ranging from 3.48 to 5.15, MAF ranging from 0.08 to 0.34, and the marker chr_16_47690 explained the highest phenotypic variance (43%) (Table 3 and Fig. 6).

**Fig. 7:**
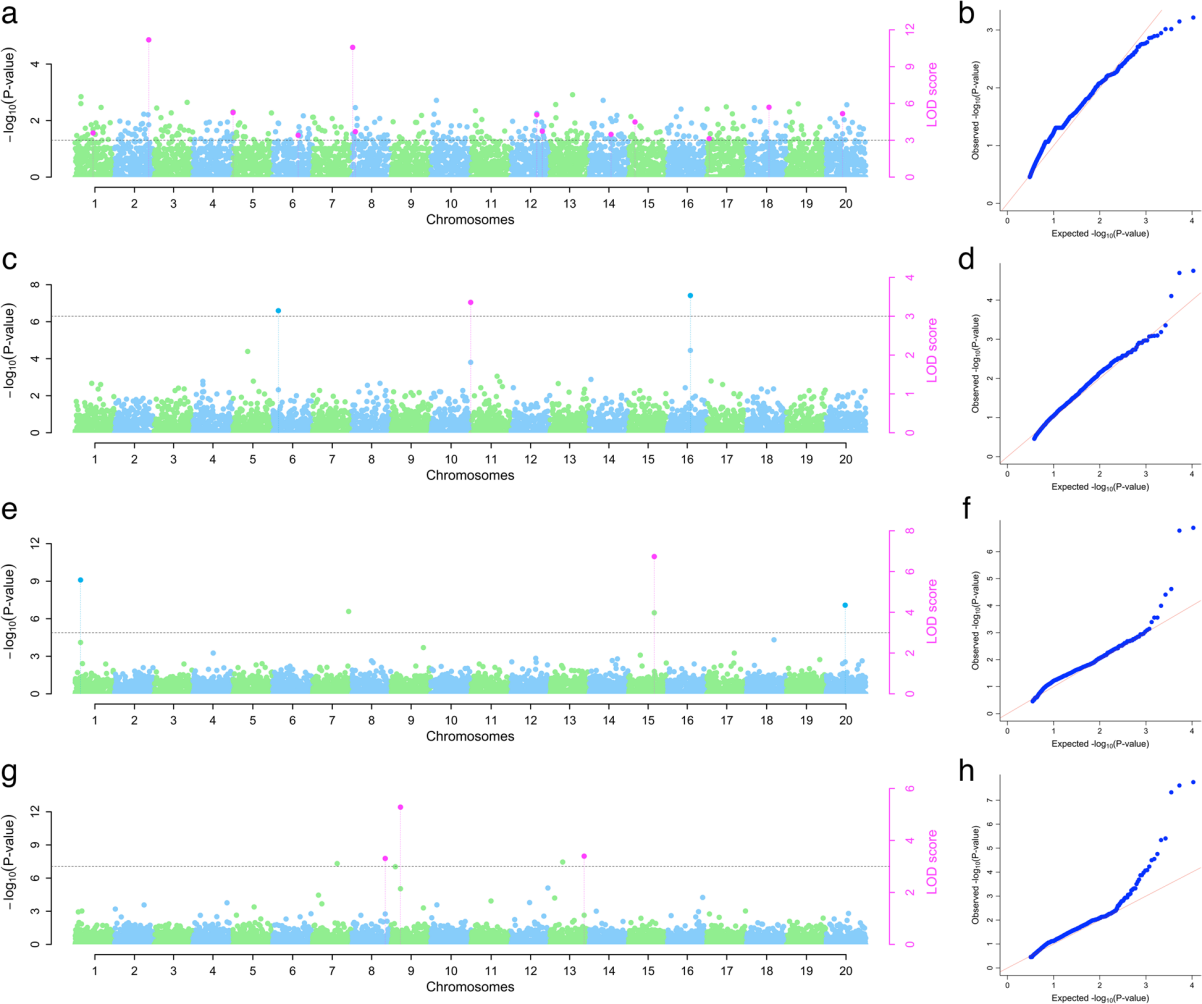
Genome-wide association analysis of plant vigor (vigor), yam anthracnose severity (YAD), yam mosaic severity (YMD), and tuber yield per plant (yield) for combined evaluation period of 2021 and 2022. Manhattan and QQ plots indicating SNPs associated with the vigor (5a and b), YAD (5c and d), YMD (5e and f), and yield (5 g and h). The y-axis represents the p-value of the marker-trait association on a –log10 scale

The combined analysis revealed seven significant SNP markers on chromosomes 12, 14, 15, 17, 18, and 20, with LOD values ranging from 3.13 to 5.69, MAF ranging from 0.11 to 0.48, and the marker chr_12_31668 explained the highest phenotypic variance (23%). For anthracnose severity, five significant SNP markers were found on chromosomes 6, 10, and 16 with LOD values ranging from 3.14 to 3.75. MAF ranging from 0.11 to 0.28, and the marker chr_16_14899 explained the highest phenotypic variance (11%). For mosaic severity, five significant SNP markers were found on chromosomes 1, 15, and 20 with LOD values ranging from 4.35 to 6.73, MAF ranging from 0.16 to 0.21, and the marker chr_15_19801explained the highest phenotypic variance (56%). For tuber yield, three significant SNP markers were found on chromosomes 8, 9, and 13 with LOD values ranging from 3.31 to 5.28, MAF ranging from 0.08 to 0.14, and the marker chr_9_3704 explained the highest phenotypic variance (42%) (Table 3 and Fig. 7).

#### Identification of existing putative genes

Of the 13 GWAS hits found for plant vigor, seven SNP markers were identified around the vicinity of some important genes on the yam reference genome. The GWAS hit on chromosome 2 is located on the genome region harboring the Cytochrome P450 (Cyt_P450); on chromosome 6, harboring the MCM OB domain (MCM_OB); on chromosome 8, harboring FBD domain (FBD); on chromosome 12, harboring Vps16 C-terminal (Vps16_C) and Fructose-1–6-bisphosphatase class I, N-terminal (FBPase_N); on chromosome 14, harboring Sugar phosphate transporter domain (Sugar_P_trans_dom) and UAA transporter family (UAA); and on chromosome 18, harboring Reverse transcriptase (RVT_2) (Table 4).

**Table 4:** Candidate genes within chromosomic regions associated with plant vigor, yam anthracnose severity, yam mosaic severity and tuber yield

Trait	SNP marker	Putative gene	Putative function
Plant vigor	chr_2_41285	Cyt_P450	Regulates brassinosteriod biosynthesis and increases vegetative growth in plants [13]
	chr_8_1451	FBD	Precise function unknown
	chr_12_31668	Vps16_C	Essential for vacuolar protein sorting for viability in plants [14]
	chr_12_19414	FBPase_N	Enhance water uptake capacity through enhanced root vigor [15]
	chr_14_13604	Sugar_P_trans_dom and UAA	Influences the cell metabolism, microtube stabilization, and cell shape in the root of the plant [16]
Yam anthracnose severity	chr_10_130470	Epimerase_deHydtase	Plays vital role in cell surface properties and virulence in plants. The gene affects cell surface properties, virulence, and extracellular enzyme production [17]
	chr_16_14899	NB-ARC	Involved in pathogen recognition and subsequent activation of innate immune responses and consists of three subdomains: NB, ARC1, and ARC2 [18]
		Rx N-terminal	Convey plant disease resistance against pathogens by producing R proteins [19]
Yam mosaic severity	chr_15_19801	RNaseH_domain	Mediate viral and cellular replication and antiviral defense in eukaryotes and prokaryotes, splicing, and DNA repair [20]
Tuber yield	chr_8_39074	GNK2	Inhibits the growth of phytopathogenic fungi and enhance protection against environmental stress in plant [21]
	chr_9_3704	CCT_CS	Auto regulatory response of photoperiodic flowering, associated with biomass and grain yield, photosynthesis, nutrition use efficiency and stress tolerance [22]
		Jas motif	Essential roles in response to tissue wounding, repairing damage, and enhancing germination and development in plants [23]
	chr_13_43659	Fum_Rdtase/Succ_DH_flav-like_C	Essential in plant respiratory mechanism [24]
		Cyt_P450	Regulates brassinosteriod biosynthesis and increases vegetative growth in plants [13]

For YAD, the identified SNP marker on chromosome 10 harbors the NAD-dependent epimerase/dehydratase (Epimerase_deHydtase) while the SNP marker on chromosome 16 harbors the NB-ARC domain (NB-ARC) and the Rx N-terminal domain (Rx N-terminal). For YMD, the identified SNP marker on chromosome 15 harbors the Ribonuclease H domain (RNaseH_domain) (Table 4).

For tuber yield, the associated SNP marker on chromosome 8 harbors the Gnk2-homologous domain (Ginkbilobin-2-GNK2), on chromosome 9 harbors the CO/COL/TOC1 (CCT_CS) and the Jas motif, and the associated SNP marker on chromosome 13 harbors Fumarate reductase/succinate dehydrogenase flavoprotein-like, C-terminal (Fum_Rdtase/Succ_DH_flav-like_C) and Cytochrome P450 (Cyt_P450) (Table 4).

## Discussion

### Phenotypic variability

The existing natural variability among the accessions for the traits under consideration was high and very informative. The high broad-sense heritability of 69% for plant vigor, 74% for anthracnose severity, 84% for mosaic virus severity, and 74% for tuber yield per plant,demonstrated the possibility for high response to selection. As a rule, traits with high heritability estimates can be modified more easily by selection and breeding than traits with lower heritability [25]. In addition, the observed genetic variation in the study materials indicates their relevance for yam genetic studies in DRC.

### Population differentiation

The knowledge of population structure within the panel of yam accessions used for this study is important to ensure the correction for spurious associations between markers and traits in GWAS analysis. The population structure of the present study based on the delta K revealed 6 as the optimal sub-populations. Though low level of admixture exists in the germplasm, two accessions found in *D. cayenensis*, and *D. dumetorum*, and nine accessions of *D. praehensilis* could be explained as the possible inclusion of the progeny of these species resulting from few generations of hybridization into the germplasm. However, for *D. rotundata,* the eight accession found as admixt means the genome is yet to achieve fixation as *D. rotundata* has been reported as a hybrid of *D. praehensilis* and *D. abyssinica* [26, 27]. The high genetic variability is an indication of the potentials of the studied accessions for genetic improvement with consideration for improved plant vigor, yam anthracnose disease resistance, yam mosaic disease resistance, and improved tuber yield. The phylogeny analysis revealed similar number of cluster (six) as the population structure analysis, indicating their relevance in preventing spurious associations in GWAS [12, 28].

### Marker-traits association and identification of putative genes

The whole-genome scan for phenotypic and allelic variation in plant vigor, yam anthracnose disease resistance, yam mosaic disease resistance, and tuber yield identified 22 genomic regions on 15 chromosomes with significant LOD score (≥ 3). In the mixed model, and to correct false-positive associations, we made used of both the Q factor representing the population structure and the K matrix representing the kinship. A total of 13 SNP markers were associated with plant vigor, three SNP markers with anthracnose disease resistance, three SNP markers with mosaic disease resistance, and three SNP markers with tuber yield that could be of importance in the implementation of marker-informed selection for these traits. Previous studies have also reported GWAS hits on some chromosomes where this study has also found significant marker-trait associations.

For plant vigor, there has been no report hitherto on the genomic regions associated with plant vigor in any yam species however, this study found 13 SNPs distributed across 11 chromosomes. Of these 13 loci, four SNP loci have been found to harbor genes (Cyt_P450; Vps16_C; FBPase_N; Sugar_P_trans_dom) that play essential role in enhancing vegetative growth in plants [13], viability of plant root [14] and enhancing root vigor for water uptake [15].

For yam anthracnose disease resistance, Agre et al. [29] reported significant SNP association on chromosomes 7, 15, and 18 from where a total of five genes were found around the SNPs loci in *D. alata*. This study found two more SNP loci on chromosomes 10 and 16 (Epimerase_deHydtase; NB-ARC; Rx N-terminal) with essential roles in affecting the cell surface properties, virulence and extracellular enzyme production [17], pathogen recognition and activation of innate immune responses [18], and production of R-proteins to convey resistance to plant diseases [19].

For yam mosaic disease resistance, significant SNP marker have been reported on chromosome 15 alongside other four chromosomes from where several genes that are essential in plant defense mechanism and plant growth were found around the vicinity of the identified markers in *D. rotundata* [10]. This study also found significant SNP on chromosome 15 harboring the RNaseh_domain with essential function in antiviral defense mechanism in plant [20].

For tuber yield, significant SNP have been reported on chromosome 8 alongside eight other chromosomes from where two genes (AUX/IAA protein and Glycine-rich protein) have been identified around the vicinity of the identified markers in *D. rotundata* [10]. This study also found significant SNP on chromosome 8 as well as two other SNP loci on chromosomes 9 and 13 harboring genes (GNK2, CCT_CS, Jas motif, Fum_Rdtase/Succ_DH_flav-like_C, and Cyt_P450) that are essential for plant defense mechanism, plant growth and development, photo-assimilates production and partitioning around the vicinity of the SNPs loci.

## Conclusion

In DRC, useful genetic variability exists in the panel of 182 yam accessions considered for this study. The genetic architecture of plant vigor, YAD, YMD, and plant yield are regulated by various SNPs unevenly distributed across the 20 chromosomes of the yam species used for this study. The associated SNP markers with plant vigor, YAD, YMD, and tuber yield could offer some potentials for employment for targeted and accelerated vigor, mosaic virus and anthracnose resistance, and tuber yield per plant in the species of yam considered for this study. The information from this study could help design new breeding strategies to capture superior alleles for improved vigor in yam, mosaic virus and anthracnose disease resistance and tuber yield per plant in future marker-based breeding in DRC.

## Materials and method

### Experimental site and planting materials

A panel of 182 yam accessions distributed across six species of yam (Sup. Table S1) obtained from previous germplasm collection exercise [4] were used for this study. The panel of yam accessions were evaluated for two years (2021 and 2022) at the University of Kisangani research terrain (longitude 0°33′05.9"N, latitude 25°05′17.3"E, Altitude 396 m a.s.l, Elevation 397 m a.s.l). The evaluation site is characterized by dense humid forest vegetation with an irregularly distributed rainfall pattern throughout the year (3156 mm annual). The soil type is mostly oxisols (ferralsols according to FAO classification) [30] and a mean temperature range of 21–35 °C minimum and maximum temperatures, respectively.

### Phenotypic data collection

The accessions were planted using 12 by 16 lattice design with two replicates. Experimental plot consists of five plants on five-meter ridge spaced at 1 m within and between plants. The 182 accessions were phenotyped for two planting seasons. Tuber yield, plant vigor, YMD and YAD were assessed according to the recommendations of Asfaw [31] and yam crop ontology https://yambase.org/tools/onto/ (access on 20^th^ November 2022). Genotype fresh weight per plant was considered as yield per plant. Plant vigor, YMD, and YAD assessment were described in Table 5. The area under the disease progression curve (AUDPC), a valuable quantitative summary of disease severity for YMD and YAD over time was estimated using the trapezoidal method [32]. This method discretizes the time variable and calculates the average disease severity between each pair of adjacent time periods:

$$AUDPC=\sum\limits_{i=1}^{N}\frac{({Y}_{i}+ {Y}_{i+1})}{2} ({t}_{i+1}- {t}_{i})$$

*where N is the number of assessments made, Y*_i_* is the anthracnose or virus severity score on date i, and t is the time in days between assessments Y*_i_* and Y*_i + 1_.

**Table 5:** Assessment of plant vigor, yam mosaic and anthracnose diseases severity

S/N	Trait	Nature of the trait	Collection period	Collection method
1	Plant vigor	Visual assessment of the vigor ofthe vine and leaves of the new plant in a plot	4 MAS	Using a 1–3 scale where 1 = weak (75% of the plants or all the plants in a plot are small and have few leaves and thin vines), 2 = medium (intermediate or normal), and 3 = vigorous (75% of the plants or all the plants in a plot are robust, with thick vines and leaves very well developed or with abundant foliage)
2	YMD severity	Visual assessment of the grade of reaction of the plant to the viruses infection, varying from mottle, mosaics until total leaves deformation recording of the severity as a proportion or percentage of plant surface affected	2 – 6 MAS	Using a visual five ordinal scale (1–5 scale), where 1 = no visible symptoms, 2 = mosaic on few leaves, symptom recovery over time, 3 = mild symptoms on many leaves but no leaf distortion, 4 = severe mosaic on most leaves, leaf distortion, and 5 = severe mosaic (bleaching), severe leaf distortion and stunting
3	YADS severity	Visual assessment of anthracnose severity by observing the relative or absolute area of plant tissue affected by yam anthracnose disease and recording of the severity as a proportion or percentage of plant surface affected	2 – 6 MAS	using a visual 1–5 general scale, where 1 = No visible symptoms of anthracnose disease, 2 = Few anthracnose spots or symptoms on 1 to ~ 25% of the plant, 3 = Anthracnose symptoms covering ~ 26 t0 ~ 50% of the plant, 4 = Symptom on > 51% of the plant, and 5 = Severe necrosis and death of the plant

*MAS* Months after sprouting

### Genotyping

Leaf samples were collected over 20 g of silica gel in covered plastic containers and kept under dark condition at room temperature for one week for adequate drying. Dried yam leaves were sent to Bioscience-IITA, Ibadan Nigeria where gDNA was extracted using CTAB protocol with slight modification. DNA quality was assessed using nanodrop before sending to Diversity Array Technology (DArT) Ltd Pty., Canberra, Australia for sequencing. High-throughput genotyping was conducted in 96 plex DArTseq protocol, and SNPs were called using the DArT’s proprietary software DArTSoft, as described by Killian et al. [33]. Generated reads was aligned with the *D. rotundata* reference genome V.2 [26].

### Phenotypic data analysis

Analysis of variance (ANOVA) was conducted through mixed linear model using lmerTest package in R [21] by considering genotype as fixed effect while year, rep and block were considered as random effects as described in the model below.

$$Y_{ijkl}=\mu+G_i+Rep_j+Rep{\left(Blk\right)}_{j(k)}+Y_l+G\times Y_{(il)}+e_{ijkl}$$

*where Y*_ijk_* is the phenotypic performance of accession for traits under consideration, µ is the average accession performance, G*_i_* is the effect of accession i, Rep*_j_* is the effect of replication j, Rep(Blk)*_j(k)_*is the block k effect nested in replication j, Y*_l_* is the effect of year l, G × Y *_(il)_* is the effect of the accession i by year l interaction, and e*_ijkl_* is the residual effect.*

Degrees of relationship among the assessed traits was determined using the Pearson’s correlation coefficient and visualized using ggpairs function in ggally package [34]. Broad-sense heritability (H^2^), phenotypic coefficient of variance (PCV), and genotypic coefficient of variance (GCV) were calculated using the values derived from the respective variance components. H^2^ was classified as low (< 30%), medium (30–60%), and high (> 60%), according to Johnson et al. [35]. Following Deshmukh et al. [36], PCV and GCV estimates that were greater than 20% were rated as high, between 10 and 20% were rated as medium and lower than 10% were regarded as low.

$$H2= \frac{{\updelta }_{\mathrm{g}}^{2}}{{\updelta }_{\mathrm{g}}^{2}+\frac{{\updelta }_{\mathrm{gl}}^{2}}{\mathrm{l}} +\frac{{\updelta }_{\mathrm{e}}^{2}}{\mathrm{rl}}} \times 100= \frac{{\updelta }_{\mathrm{g}}^{2}}{{\updelta }_{\mathrm{g}}^{2}+{\updelta }_{\mathrm{E}}^{2}} \times 100 = \frac{{\updelta }_{\mathrm{G}}^{2}}{{\updelta }_{\mathrm{P}}^{2}} \times 100$$

$$PCV=(\frac{\surd {\updelta }_{\mathrm{P}}^{2}}{\upmu }) \times 100$$

$$GCV=(\frac{\surd {\updelta }_{\mathrm{g}}^{2}}{\upmu }) \times 100$$

*where; δ*^*2*^*p* = *phenotypic variance, δ*^*2*^*g* = *genotypic variance, δ*^*2*^*gl* = *genotype by year interaction variance; δ*^*2*^*e: residual variance, r* = *number of replication; l* = *number of years; µ: grand mean of the trait.*

## Genotypic data analysis

Multiple sequences were generated by the DArTSeq platform using proprietary analytical pipelines. The HapMap file received from the DArT platform was converted into a variant call format. A total of 20,275 SNP markers were identified from the raw data and after filtering with VCFtools (Danecek et al., 2011) for minor allele frequency (MAF (0.05)), read depth (> 5), missing rate (80%), Genotype Quality (GQ = 20), maximum and minimum allele = 2 and no indels. A total of 11,721 SNP markers were retained after the filtering for downstream analysis. Summary statistics such as MAF, polymorphism information content (PIC), observed and expected heterozygosity (OH/EH) using PLINK 2 [37].

### Genetic diversity and population structure analysis

Genetic diversity among the accessions and population structure was assessed using three methods namely; model-based maximum likelihood estimation of ancestral subpopulations using admixture [38], the phylogeny analysis through analysis of phylogenetic and evolution (APE) package [39] while the dendrogram was plotted using the ggtree package [40], PCA through FactorMiner R package [41] in R. Structure simulations were carried out using a burn-in period of 20,000 iterations and a Markov chain Monte Carlo (MCMC) set at 20,000. A binary file was generated using PLINK and subjected to cross-validation approaches for determination of the optimal K value. A cut-off value of 50% was applied and used to estimate membership probabilities. Genotypes were then assigned to groups accordingly. Population structure was further plotted using bar plot function implemented in R. For the PCA, the optimal number of clusters was assessed using the “silhouette” function implemented in FactoMiner R package [29].

### Traits association analysis and gene annotation

A mixed linear model implemented in the GAPIT package in R was used to compute associations using the mixed model [42].

$$\mathrm{y }=\mathrm{ Xb }+\mathrm{ Zu }+\mathrm{ e}$$

*where y is the vector of the phenotypic observations, X represents the SNP markers (fixed effect), Z represents the random kinship (co-ancestry) matrix, b is a vector representing the estimated SNP effects, u is a vector representing random additive genetic effects, and e is the vector for random residual errors.*

A co-ancestry matrix from ADMIXTURE and kinship were included as covariates in the mixed-linear model using the Multi-random mixed linear model (MrMLM) respectively, to reduce spurious associations. The traits association analysis was conducted using single (year based) and combined BLUEs. The significant SNP markers were detected using Bonferroni threshold as stated by Cheng et al. [43] through six different genetic models namely: multi-locus random-SNP-effect Mixed Linear Model [44]; Fast multi-locus random-SNP-effect EMMA (FASTmrEMMA) [45]; Iterative Sure Independence Screening EM-Bayesian LASSO (ISIS EMBLASSO) [46]; polygenic-background-control- based least angle regression plus empirical Bayes (pLARmEB) [47]; polygenic- background-control-based KruskalWallis test plus empirical Bayes (pKWmEB) [48]; and fast mrMLM (FASTmrMLM) [46]. Quantile–quantile (QQ) plots were generated by plotting the negative logarithms (− log10) of the *p*-values against their expected *p*-values to fit the appropriateness of the GWAS model with the null hypothesis of no association and to determine how well the models accounted for population structure.

The possible candidate genes within the significant QTL region were searched in the defined range window of 1 MB at 500 Kb (downstream and upstream) from the yam Generic File Format (GFF3) file. Using the Generic File Format of the yam reference genome [26], the genes ID in the generic region were identified. Functions of the different putative genes were accessed using public database such as Interpro [49] and European Molecular Biology Laboratory-European Bioinformatics Institute (EMBL-EBI) [50].

## Supplementary Information

**Additional file 1. **

## Data Availability

Data can be obtained upon request from the corresponding author. VCF data available on the www.yambase.org
