# Supplementary material for: Association mapping in multiple yam species (Dioscorea spp.) of quantitative trait loci for yield-related traits
Source: BMC Plant Biol. 2023 Jul 11;23:357. doi: 10.1186/s12870-023-04350-4 (PMC10334582; doi:10.1186/s12870-023-04350-4)
Supplement: Supplementary file 1 — Additional file 1. [file 12870_2023_4350_MOESM1_ESM.docx]

**Supplementary results**

Sup. figure 1: Optimal number of cluster from the Bayesian Information Criteria (left) indicated K = 6 and scree plot for potential groups (right) also showed six potential groups


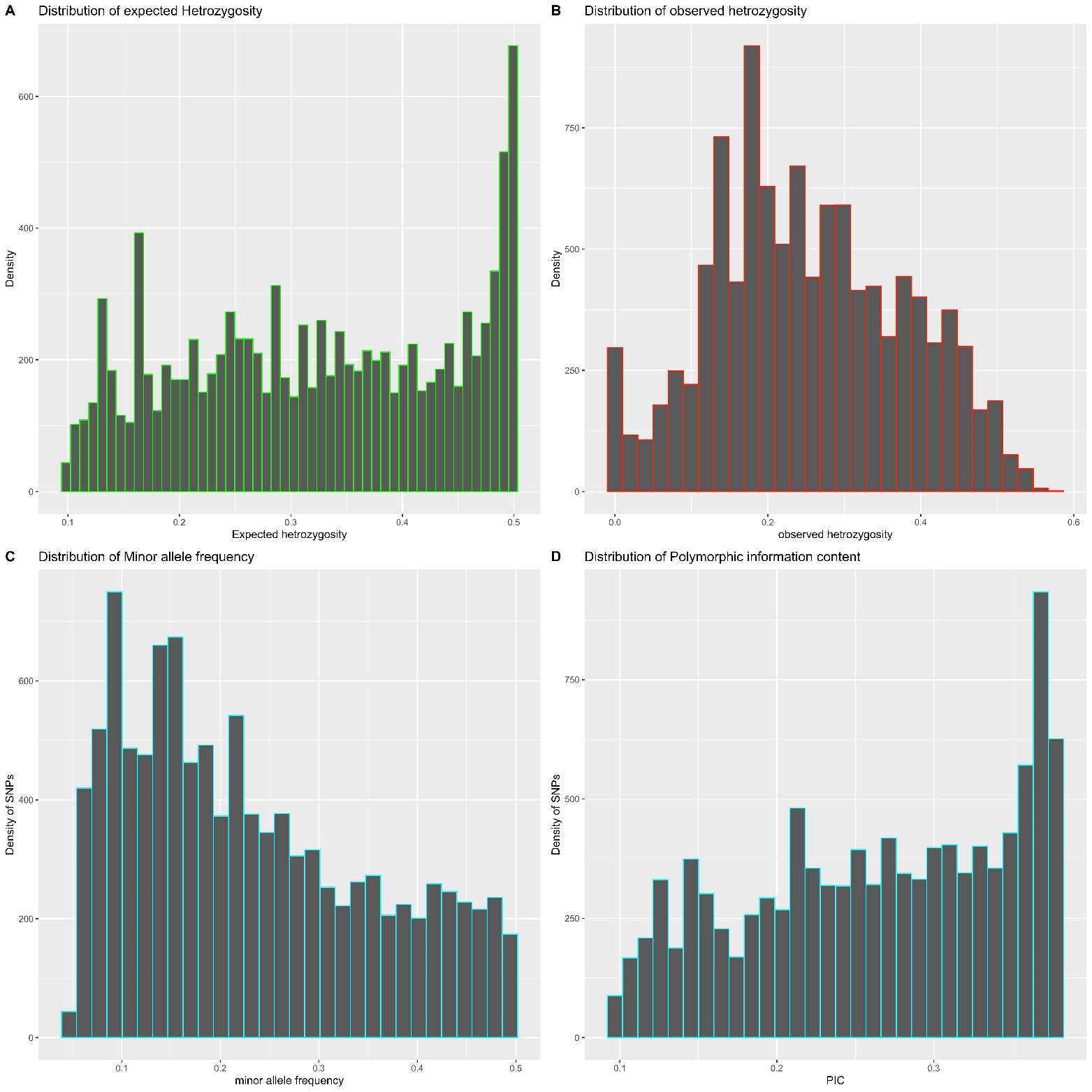


Sup. figure 2: Distribution of expected heterozygosity (A); distribution of observed heterozygosity (B); distribution of minor allele frequency (C); and distribution of polymorphic information content (D)

Sup. Table 1: Population structure at cluster k=5, membership and admixture level

| Geno | CV1 | CV2 | CV3 | CV4 | CV5 | CV6 | cluster |
| --- | --- | --- | --- | --- | --- | --- | --- |
| TDa21_032 | 0.999948 | 0.00001 | 0.00001 | 0.00001 | 0.000012 | 0.00001 | 1 |
| TDa21_014 | 0.999948 | 0.00001 | 0.00001 | 0.00001 | 0.000012 | 0.00001 | 1 |
| TDb21_130 | 0.999949 | 0.00001 | 0.00001 | 0.00001 | 0.000011 | 0.00001 | 1 |
| TDa21_003 | 0.999941 | 0.00001 | 0.000019 | 0.00001 | 0.00001 | 0.00001 | 1 |
| TDa21_132 | 0.999941 | 0.00001 | 0.000019 | 0.00001 | 0.00001 | 0.00001 | 1 |
| TDb21_076 | 0.999949 | 0.00001 | 0.000011 | 0.00001 | 0.00001 | 0.00001 | 1 |
| TDa21_034 | 0.99995 | 0.00001 | 0.00001 | 0.00001 | 0.00001 | 0.00001 | 1 |
| TDa21_152 | 0.99995 | 0.00001 | 0.00001 | 0.00001 | 0.00001 | 0.00001 | 1 |
| TDa21_050 | 0.99995 | 0.00001 | 0.00001 | 0.00001 | 0.00001 | 0.00001 | 1 |
| Tda21_150 | 0.99995 | 0.00001 | 0.00001 | 0.00001 | 0.00001 | 0.00001 | 1 |
| Tda21_192 | 0.99995 | 0.00001 | 0.00001 | 0.00001 | 0.00001 | 0.00001 | 1 |
| TDb21_023 | 0.99995 | 0.00001 | 0.00001 | 0.00001 | 0.00001 | 0.00001 | 1 |
| TDa21_189 | 0.00001 | 0.843535 | 0.00001 | 0.00001 | 0.00001 | 0.156425 | 2 |
| TDa21_169 | 0.00001 | 0.511187 | 0.00001 | 0.488773 | 0.00001 | 0.00001 | 2 |
| TDa21_064 | 0.00001 | 0.516585 | 0.00001 | 0.483375 | 0.00001 | 0.00001 | 2 |
| TDb21_022 | 0.00001 | 0.516585 | 0.00001 | 0.483375 | 0.00001 | 0.00001 | 2 |
| TDa21_068 | 0.00001 | 0.519839 | 0.00001 | 0.480121 | 0.00001 | 0.00001 | 2 |
| TDb21_086 | 0.00001 | 0.519839 | 0.00001 | 0.480121 | 0.00001 | 0.00001 | 2 |
| TDa21_098 | 0.00001 | 0.524989 | 0.00001 | 0.474971 | 0.00001 | 0.00001 | 2 |
| TDa21_005 | 0.00001 | 0.527003 | 0.00001 | 0.472957 | 0.00001 | 0.00001 | 2 |
| TDa21_084 | 0.00001 | 0.527078 | 0.00001 | 0.472882 | 0.00001 | 0.00001 | 2 |
| TDa21_173 | 0.00001 | 0.539513 | 0.00001 | 0.460447 | 0.00001 | 0.00001 | 2 |
| TDa21_008 | 0.00001 | 0.540478 | 0.00001 | 0.459482 | 0.00001 | 0.00001 | 2 |
| TDa21_120 | 0.00001 | 0.5692 | 0.00001 | 0.43076 | 0.00001 | 0.00001 | 2 |
| TDa21_072 | 0.00001 | 0.999943 | 0.00001 | 0.000017 | 0.00001 | 0.00001 | 2 |
| TDa21_042 | 0.00001 | 0.999943 | 0.00001 | 0.000017 | 0.00001 | 0.00001 | 2 |
| TDa21_019 | 0.00001 | 0.999943 | 0.00001 | 0.000017 | 0.00001 | 0.00001 | 2 |
| TDb21_002 | 0.00001 | 0.999943 | 0.00001 | 0.000017 | 0.00001 | 0.00001 | 2 |
| TDa21_125 | 0.00001 | 0.999944 | 0.00001 | 0.000016 | 0.00001 | 0.00001 | 2 |
| TDa21_009 | 0.00001 | 0.999947 | 0.00001 | 0.000013 | 0.00001 | 0.00001 | 2 |
| TDa21_133 | 0.000011 | 0.999946 | 0.00001 | 0.000013 | 0.00001 | 0.00001 | 2 |
| TDa21_095 | 0.00001 | 0.99995 | 0.00001 | 0.00001 | 0.00001 | 0.00001 | 2 |
| TDa21_180 | 0.00001 | 0.99995 | 0.00001 | 0.00001 | 0.00001 | 0.00001 | 2 |
| TDa21_160 | 0.00001 | 0.99995 | 0.00001 | 0.00001 | 0.00001 | 0.00001 | 2 |
| TDa21_144 | 0.00001 | 0.99995 | 0.00001 | 0.00001 | 0.00001 | 0.00001 | 2 |
| TDa21_149 | 0.000011 | 0.999949 | 0.00001 | 0.00001 | 0.00001 | 0.00001 | 2 |
| TDa21_073 | 0.000011 | 0.999949 | 0.00001 | 0.00001 | 0.00001 | 0.00001 | 2 |
| TDr21_021 | 0.00001 | 0.00001 | 0.974584 | 0.00001 | 0.025376 | 0.00001 | 3 |
| TDr21_007 | 0.00001 | 0.00001 | 0.999944 | 0.00001 | 0.000016 | 0.00001 | 3 |
| TDr21_092 | 0.00001 | 0.00001 | 0.999947 | 0.00001 | 0.000013 | 0.00001 | 3 |
| TDr21_015 | 0.00001 | 0.00001 | 0.99995 | 0.00001 | 0.00001 | 0.00001 | 3 |
| TDr21_046 | 0.00001 | 0.00001 | 0.99995 | 0.00001 | 0.00001 | 0.00001 | 3 |
| TDr21_158 | 0.00001 | 0.00001 | 0.99995 | 0.00001 | 0.00001 | 0.00001 | 3 |
| TDr21_131 | 0.00001 | 0.00001 | 0.99995 | 0.00001 | 0.00001 | 0.00001 | 3 |
| TDr21_177 | 0.00001 | 0.00001 | 0.99995 | 0.00001 | 0.00001 | 0.00001 | 3 |
| TDr21_179 | 0.00001 | 0.00001 | 0.99995 | 0.00001 | 0.00001 | 0.00001 | 3 |
| TDr21_085 | 0.00001 | 0.00001 | 0.99995 | 0.00001 | 0.00001 | 0.00001 | 3 |
| TDr21_186 | 0.00001 | 0.00001 | 0.99995 | 0.00001 | 0.00001 | 0.00001 | 3 |
| TDr21_033 | 0.00001 | 0.00001 | 0.99995 | 0.00001 | 0.00001 | 0.00001 | 3 |
| TDr21_047 | 0.00001 | 0.00001 | 0.99995 | 0.00001 | 0.00001 | 0.00001 | 3 |
| TDr21_181 | 0.00001 | 0.00001 | 0.99995 | 0.00001 | 0.00001 | 0.00001 | 3 |
| TDr21_154 | 0.00001 | 0.00001 | 0.99995 | 0.00001 | 0.00001 | 0.00001 | 3 |
| TDr21_108 | 0.00001 | 0.00001 | 0.99995 | 0.00001 | 0.00001 | 0.00001 | 3 |
| TDr21_045 | 0.00001 | 0.00001 | 0.99995 | 0.00001 | 0.00001 | 0.00001 | 3 |
| TDr21_066 | 0.00001 | 0.00001 | 0.99995 | 0.00001 | 0.00001 | 0.00001 | 3 |
| TDr21_119 | 0.00001 | 0.00001 | 0.99995 | 0.00001 | 0.00001 | 0.00001 | 3 |
| TDr21_083 | 0.00001 | 0.00001 | 0.99995 | 0.00001 | 0.00001 | 0.00001 | 3 |
| TDr21_162 | 0.00001 | 0.00001 | 0.99995 | 0.00001 | 0.00001 | 0.00001 | 3 |
| TDr21_043 | 0.00001 | 0.00001 | 0.99995 | 0.00001 | 0.00001 | 0.00001 | 3 |
| TDr21_067 | 0.00001 | 0.00001 | 0.99995 | 0.00001 | 0.00001 | 0.00001 | 3 |
| TDr21_027 | 0.00001 | 0.00001 | 0.99995 | 0.00001 | 0.00001 | 0.00001 | 3 |
| TDr21_111 | 0.00001 | 0.00001 | 0.99995 | 0.00001 | 0.00001 | 0.00001 | 3 |
| TDr21_060 | 0.000011 | 0.00001 | 0.999949 | 0.00001 | 0.00001 | 0.00001 | 3 |
| TDr21_128 | 0.00001 | 0.00001 | 0.00001 | 0.507086 | 0.00001 | 0.492874 | 4 |
| TDr21_101 | 0.00001 | 0.00001 | 0.00001 | 0.507785 | 0.00001 | 0.492175 | 4 |
| TDr21_020 | 0.00001 | 0.00001 | 0.00001 | 0.512767 | 0.00001 | 0.487193 | 4 |
| TDr21_031 | 0.00001 | 0.00001 | 0.00001 | 0.514878 | 0.00001 | 0.485082 | 4 |
| TDr21_106 | 0.00001 | 0.00001 | 0.00001 | 0.514926 | 0.00001 | 0.485034 | 4 |
| TDr21_006 | 0.00001 | 0.00001 | 0.00001 | 0.524467 | 0.00001 | 0.475493 | 4 |
| TDr21_170 | 0.00001 | 0.00001 | 0.00001 | 0.537024 | 0.00001 | 0.462936 | 4 |
| TDr21_116 | 0.00001 | 0.00001 | 0.002038 | 0.538971 | 0.00001 | 0.458961 | 4 |
| TDr21_110 | 0.00001 | 0.04683 | 0.00001 | 0.593135 | 0.00001 | 0.360004 | 4 |
| TDr21_129 | 0.00001 | 0.18674 | 0.00001 | 0.5155 | 0.00001 | 0.29773 | 4 |
| TDr21_097 | 0.000012 | 0.00001 | 0.00001 | 0.731164 | 0.00001 | 0.268794 | 4 |
| TDr21_012 | 0.00001 | 0.00001 | 0.00001 | 0.732271 | 0.00001 | 0.267689 | 4 |
| TDr21_109 | 0.00001 | 0.00001 | 0.00001 | 0.752225 | 0.00001 | 0.247735 | 4 |
| TDr21_171 | 0.00001 | 0.00001 | 0.00001 | 0.753387 | 0.00001 | 0.246573 | 4 |
| TDr21_161 | 0.00001 | 0.128141 | 0.00001 | 0.630036 | 0.00001 | 0.241793 | 4 |
| TDr21_113 | 0.00001 | 0.125056 | 0.00001 | 0.636227 | 0.00001 | 0.238688 | 4 |
| TDr21_039 | 0.00001 | 0.177334 | 0.00001 | 0.586249 | 0.00001 | 0.236387 | 4 |
| TDr21_087 | 0.00001 | 0.179475 | 0.00001 | 0.584808 | 0.00001 | 0.235687 | 4 |
| TDr21_024 | 0.00001 | 0.200568 | 0.00001 | 0.571557 | 0.00001 | 0.227845 | 4 |
| TDr21_017 | 0.00001 | 0.204093 | 0.00001 | 0.57161 | 0.00001 | 0.224267 | 4 |
| TDr21_157 | 0.00001 | 0.00001 | 0.00001 | 0.775841 | 0.00001 | 0.224119 | 4 |
| TDr21_165 | 0.00001 | 0.129788 | 0.00001 | 0.676209 | 0.00001 | 0.193973 | 4 |
| TDr21_016 | 0.00001 | 0.177209 | 0.00001 | 0.631425 | 0.00001 | 0.191336 | 4 |
| TDr21_004 | 0.000012 | 0.209705 | 0.00001 | 0.648645 | 0.00001 | 0.141617 | 4 |
| TDr21_175 | 0.00001 | 0.20965 | 0.00001 | 0.651343 | 0.00001 | 0.138977 | 4 |
| TDr21_166 | 0.00001 | 0.05132 | 0.00001 | 0.918879 | 0.029771 | 0.00001 | 4 |
| TDr21_187 | 0.019007 | 0.00001 | 0.042477 | 0.922088 | 0.016408 | 0.00001 | 4 |
| TDr21_139 | 0.00001 | 0.00001 | 0.00001 | 0.99995 | 0.00001 | 0.00001 | 4 |
| TDr21_140 | 0.00001 | 0.00001 | 0.00001 | 0.99995 | 0.00001 | 0.00001 | 4 |
| TDr21_104 | 0.00001 | 0.00001 | 0.00001 | 0.99995 | 0.00001 | 0.00001 | 4 |
| TDr21_164 | 0.00001 | 0.000011 | 0.00001 | 0.999949 | 0.00001 | 0.00001 | 4 |
| TDr21_167 | 0.00001 | 0.00001 | 0.03373 | 0.96623 | 0.00001 | 0.00001 | 4 |
| TDr21_184 | 0.000015 | 0.225934 | 0.00001 | 0.774021 | 0.00001 | 0.00001 | 4 |
| TDr21_127 | 0.00001 | 0.295964 | 0.00001 | 0.703996 | 0.00001 | 0.00001 | 4 |
| TDr21_112 | 0.00001 | 0.465407 | 0.00001 | 0.534553 | 0.00001 | 0.00001 | 4 |
| TDr21_074 | 0.00001 | 0.466512 | 0.00001 | 0.533448 | 0.00001 | 0.00001 | 4 |
| TDa21_080 | 0.00001 | 0.49332 | 0.00001 | 0.50664 | 0.00001 | 0.00001 | 4 |
| TDa21_079 | 0.00001 | 0.497121 | 0.00001 | 0.502839 | 0.00001 | 0.00001 | 4 |
| TDr21_183 | 0.00001 | 0.00001 | 0.00001 | 0.00001 | 0.99995 | 0.00001 | 5 |
| TDr21_191 | 0.00001 | 0.00001 | 0.00001 | 0.00001 | 0.99995 | 0.00001 | 5 |
| TDr21_118 | 0.00001 | 0.00001 | 0.00001 | 0.00001 | 0.99995 | 0.00001 | 5 |
| TDr21_037 | 0.00001 | 0.00001 | 0.00001 | 0.00001 | 0.99995 | 0.00001 | 5 |
| TDr21_142 | 0.00001 | 0.00001 | 0.00001 | 0.00001 | 0.99995 | 0.00001 | 5 |
| TDr21_153 | 0.00001 | 0.00001 | 0.00001 | 0.00001 | 0.99995 | 0.00001 | 5 |
| TDr21_071 | 0.00001 | 0.00001 | 0.00001 | 0.00001 | 0.99995 | 0.00001 | 5 |
| TDr21_163 | 0.00001 | 0.00001 | 0.00001 | 0.00001 | 0.99995 | 0.00001 | 5 |
| TDr21_038 | 0.00001 | 0.00001 | 0.00001 | 0.00001 | 0.99995 | 0.00001 | 5 |
| TDr21_134 | 0.00001 | 0.00001 | 0.00001 | 0.00001 | 0.99995 | 0.00001 | 5 |
| TDr21_041 | 0.00001 | 0.00001 | 0.00001 | 0.00001 | 0.99995 | 0.00001 | 5 |
| TDr21_115 | 0.00001 | 0.00001 | 0.00001 | 0.00001 | 0.99995 | 0.00001 | 5 |
| TDr21_185 | 0.00001 | 0.00001 | 0.00001 | 0.00001 | 0.99995 | 0.00001 | 5 |
| TDr21_077 | 0.00001 | 0.00001 | 0.00001 | 0.00001 | 0.99995 | 0.00001 | 5 |
| TDr21_137 | 0.00001 | 0.00001 | 0.00001 | 0.00001 | 0.99995 | 0.00001 | 5 |
| TDr21_088 | 0.00001 | 0.00001 | 0.00001 | 0.00001 | 0.99995 | 0.00001 | 5 |
| TDr21_102 | 0.00001 | 0.00001 | 0.000011 | 0.000011 | 0.999949 | 0.00001 | 5 |
| TDr21_062 | 0.00001 | 0.00001 | 0.000011 | 0.00001 | 0.999949 | 0.00001 | 5 |
| TDr21_089 | 0.00001 | 0.000011 | 0.00001 | 0.00001 | 0.999949 | 0.00001 | 5 |
| TDr21_148 | 0.000011 | 0.00001 | 0.00001 | 0.00001 | 0.999949 | 0.00001 | 5 |
| TDr21_107 | 0.000011 | 0.00001 | 0.00001 | 0.00001 | 0.999949 | 0.00001 | 5 |
| TDr21_055 | 0.000011 | 0.00001 | 0.00001 | 0.00001 | 0.999949 | 0.00001 | 5 |
| TDr21_151 | 0.000011 | 0.00001 | 0.00001 | 0.00001 | 0.999949 | 0.00001 | 5 |
| TDr21_030 | 0.000012 | 0.00001 | 0.00001 | 0.00001 | 0.999948 | 0.00001 | 5 |
| TDr21_143 | 0.000012 | 0.00001 | 0.00001 | 0.00001 | 0.999948 | 0.00001 | 5 |
| TDr21_054 | 0.000012 | 0.00001 | 0.00001 | 0.00001 | 0.999948 | 0.00001 | 5 |
| TDr21_051 | 0.000013 | 0.00001 | 0.00001 | 0.00001 | 0.999947 | 0.00001 | 5 |
| TDr21_099 | 0.00001 | 0.00001 | 0.000013 | 0.00001 | 0.999946 | 0.00001 | 5 |
| TDr21_053 | 0.000014 | 0.00001 | 0.00001 | 0.00001 | 0.999946 | 0.00001 | 5 |
| TDr21_100 | 0.00001 | 0.000015 | 0.00001 | 0.00001 | 0.999945 | 0.00001 | 5 |
| TDr21_126 | 0.000014 | 0.00001 | 0.00001 | 0.000012 | 0.999944 | 0.00001 | 5 |
| TDr21_061 | 0.000017 | 0.00001 | 0.00001 | 0.00001 | 0.999943 | 0.00001 | 5 |
| TDd21_174 | 0.00001 | 0.00001 | 0.00001 | 0.00001 | 0.00001 | 0.99995 | 6 |
| TDd21_145 | 0.00001 | 0.00001 | 0.00001 | 0.00001 | 0.00001 | 0.99995 | 6 |
| TDd21_075 | 0.00001 | 0.00001 | 0.00001 | 0.00001 | 0.00001 | 0.99995 | 6 |
| TDd21_124 | 0.00001 | 0.00001 | 0.00001 | 0.00001 | 0.00001 | 0.99995 | 6 |
| TDd21_094 | 0.00001 | 0.00001 | 0.00001 | 0.00001 | 0.00001 | 0.99995 | 6 |
| TDd21_136 | 0.00001 | 0.00001 | 0.00001 | 0.00001 | 0.00001 | 0.99995 | 6 |
| TDd21_011 | 0.00001 | 0.00001 | 0.00001 | 0.00001 | 0.00001 | 0.99995 | 6 |
| TDd21_029 | 0.00001 | 0.00001 | 0.00001 | 0.00001 | 0.00001 | 0.99995 | 6 |
| TDd21_090 | 0.00001 | 0.00001 | 0.00001 | 0.00001 | 0.00001 | 0.99995 | 6 |
| TDc21_070 | 0.00001 | 0.00001 | 0.00001 | 0.00001 | 0.00001 | 0.99995 | 6 |
| TDc21_117 | 0.00001 | 0.00001 | 0.00001 | 0.00001 | 0.00001 | 0.99995 | 6 |
| TDc21_059 | 0.00001 | 0.00001 | 0.00001 | 0.00001 | 0.00001 | 0.99995 | 6 |
| TDc21_172 | 0.00001 | 0.00001 | 0.00001 | 0.00001 | 0.00001 | 0.99995 | 6 |
| TDc21_190 | 0.00001 | 0.00001 | 0.00001 | 0.00001 | 0.00001 | 0.99995 | 6 |
| TDc21_176 | 0.00001 | 0.00001 | 0.00001 | 0.00001 | 0.00001 | 0.99995 | 6 |
| TDc21_138 | 0.00001 | 0.00001 | 0.00001 | 0.00001 | 0.00001 | 0.99995 | 6 |
| TDc21_188 | 0.00001 | 0.00001 | 0.00001 | 0.00001 | 0.00001 | 0.99995 | 6 |
| TDc21_035 | 0.00001 | 0.00001 | 0.00001 | 0.00001 | 0.00001 | 0.99995 | 6 |
| TDc21_018 | 0.00001 | 0.00001 | 0.00001 | 0.00001 | 0.00001 | 0.99995 | 6 |
| TDp21_052 | 0.00001 | 0.00001 | 0.00001 | 0.00001 | 0.00001 | 0.99995 | 6 |
| TDp21_065 | 0.00001 | 0.00001 | 0.00001 | 0.00001 | 0.00001 | 0.99995 | 6 |
| TDp21_081 | 0.00001 | 0.00001 | 0.00001 | 0.00001 | 0.00001 | 0.99995 | 6 |
| TDp21_049 | 0.00001 | 0.00001 | 0.00001 | 0.00001 | 0.00001 | 0.99995 | 6 |
| TDp21_063 | 0.00001 | 0.00001 | 0.00001 | 0.000012 | 0.00001 | 0.999948 | 6 |
| TDp21_026 | 0.00001 | 0.00001 | 0.00001 | 0.000014 | 0.00001 | 0.999946 | 6 |
| TDd21_146 | 0.000011 | 0.00001 | 0.000011 | 0.000012 | 0.00001 | 0.999946 | 6 |
| TDd21_156 | 0.00001 | 0.00001 | 0.00001 | 0.000018 | 0.00001 | 0.999942 | 6 |
| TDd21_114 | 0.00001 | 0.00001 | 0.00001 | 0.000019 | 0.00001 | 0.999941 | 6 |
| TDc21_135 | 0.00001 | 0.372365 | 0.00001 | 0.138168 | 0.00001 | 0.489437 | Admixt |
| TDd21_069 | 0.00001 | 0.369189 | 0.00001 | 0.14186 | 0.00001 | 0.488921 | Admixt |
| TDc21_147 | 0.000011 | 0.373806 | 0.00001 | 0.139301 | 0.00001 | 0.486863 | Admixt |
| TDp21_078 | 0.00001 | 0.370405 | 0.00001 | 0.142757 | 0.00001 | 0.486809 | Admixt |
| TDd21_103 | 0.00001 | 0.369427 | 0.00001 | 0.143858 | 0.00001 | 0.486685 | Admixt |
| TDr21_141 | 0.00001 | 0.239334 | 0.00001 | 0.280511 | 0.00001 | 0.480125 | Admixt |
| TDp21_121 | 0.00001 | 0.241743 | 0.00001 | 0.278117 | 0.00001 | 0.48011 | Admixt |
| TDr21_001 | 0.00001 | 0.237133 | 0.00001 | 0.283134 | 0.00001 | 0.479702 | Admixt |
| TDr21_025 | 0.000014 | 0.237093 | 0.00001 | 0.283465 | 0.00001 | 0.479408 | Admixt |
| TDr21_096 | 0.00001 | 0.23977 | 0.00001 | 0.282253 | 0.00001 | 0.477947 | Admixt |
| TDp21_122 | 0.00001 | 0.239981 | 0.00001 | 0.282274 | 0.00001 | 0.477715 | Admixt |
| TDp21_058 | 0.00001 | 0.238756 | 0.00001 | 0.283714 | 0.00001 | 0.4775 | Admixt |
| TDr21_010 | 0.000011 | 0.228728 | 0.00001 | 0.302733 | 0.00001 | 0.468508 | Admixt |
| TDp21_159 | 0.00001 | 0.229456 | 0.00001 | 0.30244 | 0.00001 | 0.468075 | Admixt |
| TDp21_036 | 0.00001 | 0.23014 | 0.00001 | 0.303497 | 0.00001 | 0.466333 | Admixt |
| TDp21_040 | 0.00001 | 0.291244 | 0.00001 | 0.265207 | 0.00001 | 0.443518 | Admixt |
| TDp21_123 | 0.00001 | 0.290424 | 0.00001 | 0.274489 | 0.00001 | 0.435056 | Admixt |
| TDp21_182 | 0.00001 | 0.289486 | 0.00001 | 0.277001 | 0.00001 | 0.433483 | Admixt |
| TDr21_044 | 0.000018 | 0.269289 | 0.00001 | 0.479029 | 0.00001 | 0.251644 | Admixt |
| TDr21_082 | 0.000014 | 0.425181 | 0.00001 | 0.400278 | 0.00001 | 0.174507 | Admixt |
| TDr21_013 | 0.00001 | 0.46085 | 0.021827 | 0.482652 | 0.00001 | 0.03465 | Admixt |
